# Role of betel quid in changing oral pathology

**DOI:** 10.1186/1755-8166-7-S1-P5

**Published:** 2014-01-21

**Authors:** Aniket Adhikari, Subhrajyoti Mukherjee, Kaustav Roy, Ranjan Roychowdhury, Madhusnata De

**Affiliations:** 1 Department of Genetics, Vivekananda Institute of Medical Sciences, Ramakrishna Mission Seva Pratisthan, 99- Sarat Bose Road, Kolkata 700 026, West Bengal, India; 2 Department of Oral and Maxillofacial surgery of Ramakrishna Mission Seva Pratisthan, 99- Sarat Bose Road, Kolkata 700 026, West Bengal, India; 3 Department of ENT of Ramakrishna Mission Seva Pratisthan, 99- Sarat Bose Road, Kolkata 700 026, West Bengal, India

## Background

Chewing of betel quid (BQ) and areca nut is an ancient custom in South East Asia. More than 600,000,000 people chew areca nut worldwide. In India there are 75,000 to 80,000 cases of oral cancer each year and incidence rates of cancers of the oral cavity in both male and female in all urban cancers are among the highest in the world. In India, 13 -50% of the student chew BQ and pan masala frequently and prevalence rate is 14 -15% among 11-15 year of children. Areca nuts, Catechu, Slaked lime are major components of Betel quid. Nitrosamines formed from alkaloids in betel nut and reactive oxygen species (ROS) are generated due to slaked lime during betel quid chewing may be implicated in the etiology of oral cancer. Micronuclei (MN) have been proposed as a good biomarker to assess cytogenetic damage. Among the xenobiotics metabolizing enzymes, the CYP2A family is characteristics of its catalytic properties to nitrosamines. Micronuclei (MN) and CYP2A6 genetic polymorphism were studied among the Eastern and North eastern population.

## Materials and methods

In this present study subjects were screened from different camps and Department of E.N.T. & Oral and Maxillofacial surgery of RKMSP hospital, Kolkata. Exfoliated cell from the buccal mucosa were examined for micronuclei (MN). Polymorphism of CYP2A6 gene was studied from EDTA blood.

## Results

Micronuclei percentage was higher in subjects who had betel quid chewing habit. Early metabolizers are susceptible to oral cancer whereas in case of poor metabolizers chances are less.

## Conclusions

Oral pathology seems to be altered due to chewing betel quid and its ingredients with increased susceptibility to cancer.

